# Genome Sequence of the Butanol Hyperproducer *Clostridium saccharoperbutylacetonicum* N1-4

**DOI:** 10.1128/genomeA.00070-13

**Published:** 2013-03-07

**Authors:** Carlos del Cerro, Carmen Felpeto-Santero, Antonia Rojas, Marta Tortajada, Daniel Ramón, José L. García

**Affiliations:** a Environmental Biology, Centro de Investigaciones Biológicas, Consejo Superior de Investigaciones Científicas, Madrid, Spain; b Biopolis SL, Parc Científic Universitat de Valencia, Paterna, Spain; c Lifesequencing SL, Parc Científic Universitat de Valencia, Paterna, Spain

## Abstract

*Clostridium saccharoperbutylacetonicum* is one of the most important acetone-butanol-ethanol (ABE)-generating industrial microorganisms and one of the few bacteria containing choline in its cell wall. Here, we report the draft genome sequence of *C. saccharoperbutylacetonicum* strain N1-4 (6.6 Mbp; G+C content, 29.4%) and the findings obtained from the annotation of the genome.

## GENOME ANNOUNCEMENT

*Clostridium* is one of the largest bacterial genera, ranking second in size after *Streptomyces*, and members of the genus are classified as Gram-positive endospore-forming obligate anaerobes (1). Many species of *Clostridium* are of biotechnological importance, such as *Clostridium acetobutylicum*, which was used for acetone-butanol-ethanol (ABE) production during the first half of the last century before being replaced by petrochemical synthesis in the industrial production of chemicals (2). However, there has been a revival of interest in ABE fermentation, since renewable resources have become possible alternative substrates for the production of biofuels at a low cost (3). Despite the fact that *Clostridium saccharoperbutylacetonicum* has been considered a reference microorganism for ABE fermentation (4–8), it was not genetically characterized until very recently (9), and its genome remained unknown.

The genome of *C. saccharoperbutylacetonicum* N1-4 (ATCC 27021) has been sequenced using the Titanium kit and the GS-FLX pyrosequencing equipment from Roche. Preliminary assembly of raw reads was performed using Newbler software from Roche. This assembly was manually revised and improved to obtain a quality draft of 210 contigs. The genome was structurally and functionally annotated using Rapid Annotations using Subsystems Technology (RAST) (10), an automated genome annotation system, and the functions, names, and general properties of the gene products were predicted using this method. *C. saccharoperbutylacetonicum* N1-4 has one of the largest clostridial genomes (6.6 Mbp); it has a G+C content of 29.4%, encodes 20 RNAs, and contains 5,987 coding sequences.

Remarkably, *C. saccharoperbutylacetonicum* is one of the few bacteria that contain choline in the teichoic acids of their cell walls (11, 12). This property usually correlates with the expression of different modular proteins, named choline-binding proteins (CBPs), which have evolved from the fusion of a typical choline-binding domain (13) with a variety of functional protein modules that play important physiological roles (14, 15). We have annotated 66 CBPs encoded by the genome of *C. saccharoperbutylacetonicum*. At least nine of these CBPs contained functional modules showing high similarity with cell wall lytic enzymes (16).

JSpecies (17) comparison of *C. saccharoperbutylacetonicum* N1-4 and *Clostridium beijerinckii* NCIMB 8052 gives an average nucleotide identity based on BLAST (ANIb) of 78.86% (ANIb aligned 36.85%) and an average nucleotide identity based on MUMmer (ANIm) of 85.69% (ANIm aligned 20.12%). These results confirmed that although the two species share a very large number of genes, they can be taxonomically classified as different species.

### Nucleotide sequence accession number.

The *C. saccharoperbutylacetonicum* N1-4 (ATCC 27021) genome sequence has been submitted to GenBank under the accession no. AOIF00000000.
